# Supplementary material for: Prognostic Role of Circulating miRNAs in Early-Stage Non-Small Cell Lung Cancer
Source: J Clin Med. 2019 Jan 23;8(2):131. doi: 10.3390/jcm8020131 (PMC6407000; doi:10.3390/jcm8020131)
Supplement: Supplementary file 1 [file jcm-08-00131-s001.pdf]

Supplementary Material

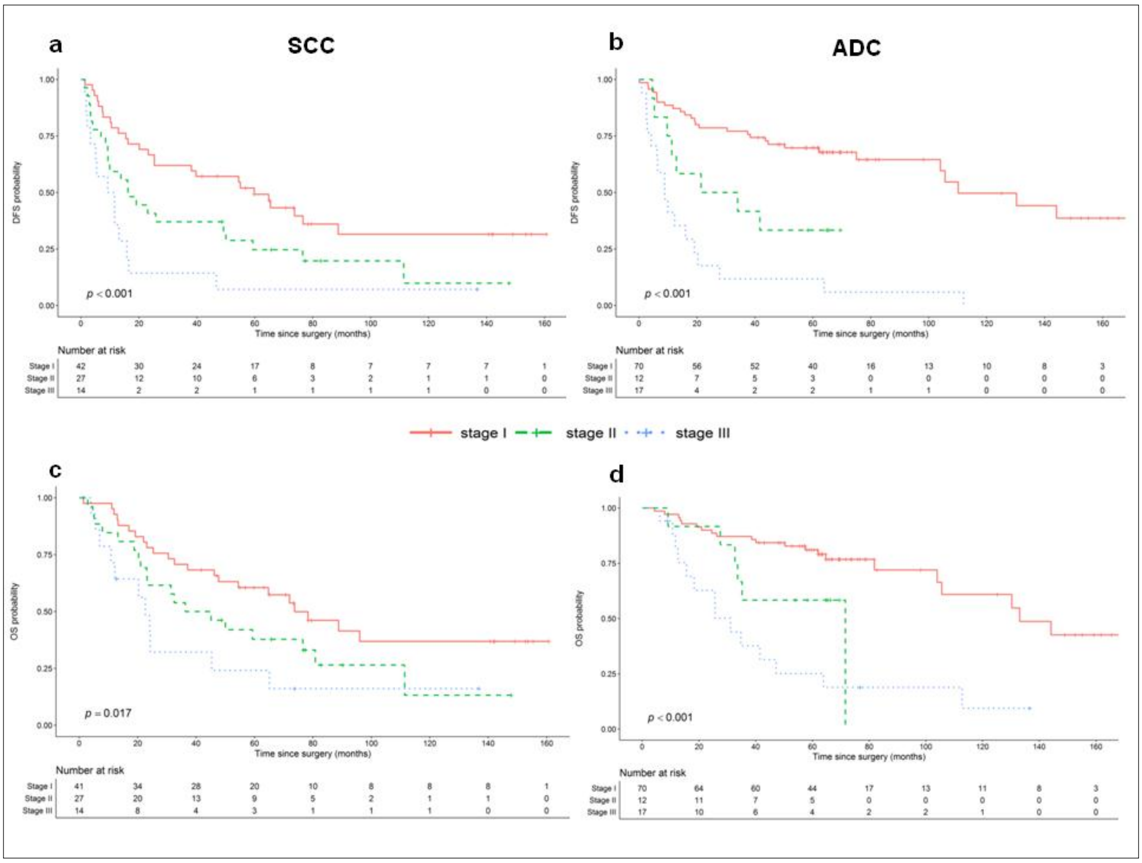

Figure S1. PFS and OS Kaplan-Meier curves for SCC patients. Progression free and overall survival risk tables and Kaplan-Meier curves for SCC (panels A and C, respectively) and ADC (panels B and D, respectively) by disease stage I (red solid line), II (green dashed line) and III (cyan dotted line); log-rank p-value for the hypothesis testing of equality of curves between disease stages are also reported.

Table S1. miRNAs significantly associated with DFS at univariable analysis for SCC patients.

Rank (j)	Name	HR	95% CI	*p* value	(j/m)×δ
1	mir-26a-5p	0.57	0.4-0.80	0.00102	0.00074
2	mir-126-3p	0.57	0.4-0.80	0.00147	0.00147
3	mir-130b-3p	0.74	0.59-0.92	0.00775	0.00221
4	mir-205-5p	1.15	1.02-1.29	0.02479	0.00294
5	mir-21-5p	0.66	0.44-0.97	0.03552	0.00368
6	mir-26b-5p	0.78	0.61-1.0	0.05318	0.00441
7	let7a-5p	0.76	0.58-1.01	0.05588	0.00515
8	mir-191-5p	0.83	0.65-1.05	0.12115	0.00588
9	mir-145-5p	1.19	0.93-1.52	0.16318	0.00662
10	mir-122-5p	0.91	0.79-1.05	0.21398	0.00735
11	mir-374a-5p	0.87	0.68-1.1	0.24146	0.00809
12	mir-103a-3p	0.87	0.69-1.1	0.24224	0.00882
13	mir-224-5p	0.9	0.75-1.08	0.26169	0.00956
14	mir-146a-5p	0.85	0.64-1.13	0.26796	0.01029
15	mir-18a-5p	0.89	0.72-1.10	0.29164	0.01103
16	mir-27a-3p	0.83	0.56-1.22	0.33141	0.01176
17	mir-17-3p	0.94	0.82-1.07	0.33518	0.0125
18	mir-100-5p	0.93	0.79-1.09	0.35798	0.01324

19	mir-31-5p	1.05	0.95-1.16	0.36864	0.01397
20	mir-93-5p	1.11	0.89-1.38	0.36984	0.01471
21	mir-143-3p	1.09	0.9-1.32	0.37828	0.01544
22	mir-34a-5p	1.06	0.93-1.21	0.38034	0.01618
23	mir-200c-3p	0.94	0.82-1.09	0.41152	0.01691
24	mir-196a-5p	1.05	0.94-1.17	0.42491	0.01765
25	mir-150-5p	0.91	0.72-1.15	0.45170	0.01838
26	mir-15b-5p	0.9	0.67-1.2	0.47132	0.01912
27	mir-10a-5p	0.94	0.8-1.11	0.47461	0.01985
28	mir-376c-3p	1.06	0.9-1.26	0.49402	0.02059
29	mir-23a-3p	0.89	0.62-1.26	0.49584	0.02132
30	mir-29a-3p	0.89	0.62-1.27	0.51856	0.02206
31	mir-19b-3p	1.07	0.87-1.33	0.52269	0.02279
32	mir-155-5p	1.04	0.92-1.18	0.52432	0.02353
33	mir-106b-5p	1.07	0.86-1.33	0.54846	0.02426
34	mir-200b-3p	0.97	0.86-1.08	0.55663	0.025
35	mir-92a-3p	0.94	0.76-1.17	0.60354	0.02574
36	mir-15a-5p	1.06	0.84-1.34	0.60957	0.02647
37	mir-124-3p	0.96	0.82-1.13	0.61049	0.02721
38	mir-296-5p	1.03	0.91-1.18	0.61123	0.02794
39	mir-75p	0.96	0.80-1.14	0.61688	0.02868
40	mir-107	0.96	0.83-1.12	0.62436	0.02941
41	mir-22-3p	1.03	0.88-1.22	0.68818	0.03015
42	mir-17-5p	0.97	0.82-1.14	0.69963	0.03088
43	mir-885-5p	0.97	0.85-1.12	0.70192	0.03162
44	mir-25-3p	0.96	0.79-1.17	0.71665	0.03235
45	mir-193a-5p	1.04	0.85-1.26	0.71690	0.03309
46	mir-128-3p	0.96	0.75-1.22	0.71741	0.03382
47	mir-19a-3p	1.03	0.86-1.24	0.72668	0.03456
48	mir-148a-3p	1.04	0.85-1.27	0.72932	0.03529
49	mir-20a-5p	1.04	0.83-1.30	0.76348	0.03603
50	mir-10b-5p	0.98	0.86-1.12	0.79229	0.03676
51	mir-30d-5p	0.97	0.73-1.27	0.80383	0.0375
52	mir-134-5p	1.01	0.89-1.15	0.82150	0.03824
53	mir-423-5p	0.97	0.75-1.25	0.82374	0.03897
54	mir-30e-5p	0.96	0.68-1.36	0.83956	0.03971
55	let7c-5p	0.98	0.81-1.18	0.84444	0.04044
56	mir-16-5p	1.02	0.83-1.24	0.86001	0.04118
57	mir-375	0.99	0.86-1.13	0.86015	0.04191
58	mir-210-3p	1.01	0.89-1.14	0.89543	0.04265
59	mir-372-3p	0.99	0.9-1.1	0.91062	0.04338
60	mir-222-3p	0.99	0.76-1.28	0.91127	0.04412
61	mir-214-3p	0.99	0.88-1.13	0.92136	0.04485
62	mir-574-3p	1.01	0.88-1.15	0.92587	0.04559
63	mir-192-5p	0.99	0.84-1.18	0.92675	0.04632
64	mir-223-3p	0.99	0.87-1.14	0.93635	0.04706
65	mir-125b-5p	0.99	0.80-1.23	0.93880	0.04779
66	mir-204-5p	1.00	0.87-1.16	0.95999	0.04853
67	mir-211-5p	1.00	0.88-1.14	0.96169	0.04926
68	mir-195-5p	1.00	0.82-1.23	0.98087	0.05

SCC: squamous cell carcinoma; HR: hazard ratio; 95% CI: 95% confidence interval; m: number of tests (68); δ: FDR level (0.05).

Table S2. miRNAs significantly associated with DFS at univariable analysis for ADC patients.

Rank (j)	Name	HR	95% CI	p-value	(j/m)×δ
1	mir-222-3p	1.37	1.06-1.76	0.01628	0.00074
2	mir-22-3p	1.23	1.03-1.49	0.02641	0.00147
3	mir-93-5p	1.41	1.02-1.99	0.03713	0.00221
4	mir-19b-3p	1.35	0.99-1.62	0.05898	0.00294
5	mir-574-3p	0.79	0.99-1.35	0.06871	0.00368
6	mir-145-5p	0.83	0.97-1.46	0.09033	0.00441
7	mir-130b-3p	1.17	0.98-1.36	0.09119	0.00515
8	mir-106b-5p	1.25	0.96-1.78	0.09365	0.00588
9	mir-29a-3p	0.87	0.95-1.70	0.10743	0.00662
10	mir-195-5p	1.15	0.95-1.61	0.11995	0.00735
11	mir-92a-3p	1.30	0.65-1.06	0.1315	0.00809
12	mir-192-5p	1.12	0.96-1.26	0.16044	0.00882
13	mir-26a-5p	1.17	0.58-1.09	0.16181	0.00956
14	mir-19a-3p	1.23	0.94-1.45	0.17002	0.01029
15	mir-10a-5p	1.26	0.73-1.07	0.19549	0.01103
16	mir-15a-5p	0.87	0.66-1.09	0.20838	0.01176
17	mir-18a-5p	0.87	0.9-1.59	0.21096	0.0125
18	mir-193a-5p	1.16	0.92-1.40	0.22952	0.01324
19	mir-107	0.89	0.78-1.07	0.25884	0.01397
20	mir-211-5p	1.31	0.77-1.07	0.26275	0.01471
21	mir-17-3p	1.22	0.93-1.32	0.26486	0.01544
22	mir-374a-5p	0.89	0.88-1.5	0.32107	0.01618
23	mir-30e-5p	0.82	0.82-1.84	0.32531	0.01691
24	mir-24-3p	0.86	0.86-1.53	0.34728	0.01765
25	mir-17-5p	0.91	0.85-1.59	0.35172	0.01838
26	mir-103a-3p	0.87	0.85-1.56	0.35288	0.01912
27	mir-16-5p	1.16	0.87-1.45	0.36709	0.01985
28	mir-10b-5p	1.15	0.78-1.10	0.37424	0.02059
29	mir-150-5p	0.93	0.76-1.11	0.39387	0.02132
30	mir-21-5p	0.84	0.63-1.21	0.41665	0.02206
31	mir-196a-5p	0.92	0.85-1.07	0.4246	0.02279
32	mir-124-3p	0.93	0.77-1.12	0.44511	0.02353
33	mir-373-3p	1.14	0.8-1.1	0.44693	0.02426
34	mir-30d-5p	1.12	0.71-1.17	0.47782	0.025
35	mir-15b-5p	1.09	0.84-1.42	0.52196	0.02574
36	mir-376c-3p	0.93	0.80-1.12	0.52692	0.02647
37	mir-885-5p	0.94	0.89-1.25	0.5349	0.02721
38	mir-204-5p	1.05	0.79-1.13	0.54496	0.02794
39	mir-122-5p	0.90	0.78-1.15	0.56932	0.02868
40	mir-25-3p	0.95	0.83-1.40	0.57534	0.02941
41	mir-375	1.06	0.81-1.13	0.57675	0.03015
42	mir-200c-3p	1.08	0.91-1.17	0.61316	0.03088
43	mir-315p	0.95	0.83-1.12	0.61771	0.03162
44	mir-296-5p	0.95	0.85-1.10	0.64285	0.03235
45	let7a-5p	1.08	0.81-1.4	0.65339	0.03309
46	mir-34a-5p	1.04	0.83-1.12	0.65611	0.03382
47	mir-27a-3p	0.95	0.66-1.29	0.65657	0.03456
48	mir-125b-5p	0.97	0.81-1.39	0.65891	0.03529
49	mir-23a-3p	0.98	0.86-1.27	0.66819	0.03603
50	mir-146a-5p	1.05	0.71-1.24	0.67203	0.03676
51	mir-26b-5p	0.98	0.73-1.24	0.70995	0.0375
52	mir-20a-5p	1.03	0.75-1.49	0.75082	0.03824
53	mir-155-5p	0.96	0.86-1.23	0.7608	0.03897
54	mir-224-5p	0.99	0.8-1.18	0.77227	0.03971
55	mir-128-3p	1.03	0.76-1.23	0.79314	0.04044

56	mir-7-5p	1.02	0.81-1.18	0.81341	0.04118
57	let7c-5p	1.02	0.78-1.22	0.83963	0.04191
58	mir-423-5p	0.98	0.77-1.24	0.84309	0.04265
59	mir-191-5p	1.02	0.80-1.30	0.85529	0.04338
60	mir-210-3p	1.02	0.85-1.15	0.86592	0.04412
61	mir-205-5p	0.99	0.87-1.12	0.87405	0.04485
62	mir-214-3p	0.99	0.84-1.16	0.88455	0.04559
63	mir-134-5p	0.99	0.86-1.14	0.88627	0.04632
64	mir-200b-3p	0.99	0.87-1.16	0.91447	0.04706
65	mir-143-3p	1.01	0.86-1.19	0.91503	0.04779
66	mir-100-5p	1.00	0.78-1.26	0.94703	0.04853
67	mir-148a-3p	1.00	0.79-1.28	0.95916	0.04926
68	mir-223-3p	0.88	0.89-1.12	0.96262	0.05

ADC: adenocarcinoma; HR: hazard ratio; 95% CI: 95% confidence interval; m: number of tests (68); δ, FDR level (0.05).

Table S3. miRNAs significantly associated with OS at univariable analysis for SCC patients.

Rank (j)	Name	HR	95% CI	*p*-value	j/m*delta
1	mir-130b-3p	0.74	0.59-0.92	0.00753	0.00074
2	mir-26a-5p	0.62	0.43-0.88	0.00767	0.00147
3	mir-126-3p	0.62	0.43-0.9	0.01242	0.00221
4	mir-205-5p	1.16	1.01-1.33	0.04138	0.00294
5	mir1915p	0.79	0.6-1.03	0.07844	0.00368
6	mir-26b-5p	0.77	0.58-1.03	0.08096	0.00441
7	let7a-5p	0.76	0.56-1.04	0.08634	0.00515
8	mir-103a-3p	0.81	0.63-1.04	0.10175	0.00588
9	mir-18a-5p	0.87	0.68-1.1	0.22993	0.00662
10	mir-143-3p	1.12	0.9-1.4	0.28962	0.00735
11	mir-21-5p	0.8	0.53-1.21	0.29074	0.00809
12	mir-31-5p	1.06	0.94-1.18	0.32981	0.00882
13	mir-122-5p	0.93	0.79-1.09	0.34096	0.00956
14	mir-22-3p	1.09	0.91-1.31	0.34951	0.01029
15	mir-211-5p	1.06	0.93-1.21	0.36452	0.01103
16	mir-100-5p	0.92	0.77-1.1	0.36987	0.01176
17	mir-155-5p	1.07	0.92-1.25	0.37383	0.0125
18	mir-196a-5p	1.06	0.93-1.2	0.37859	0.01324
19	mir-148a-3p	1.11	0.87-1.42	0.38782	0.01397
20	mir-200c-3p	0.94	0.8-1.09	0.3998	0.01471
21	mir-15a-5p	1.11	0.86-1.45	0.41335	0.01544
22	mir-17-5p	0.9	0.7-1.16	0.41578	0.01618
23	mir-150-5p	0.9	0.69-1.17	0.42221	0.01691
24	mir-27a-3p	0.85	0.55-1.3	0.44666	0.01765
25	mir-204-5p	1.06	0.91-1.24	0.47221	0.01838
26	mir-374a-5p	0.91	0.7-1.18	0.48409	0.01912
27	mir-34a-5p	1.05	0.91-1.22	0.48866	0.01985
28	mir-128-3p	0.92	0.71-1.19	0.50355	0.02059
29	mir-193a-5p	1.08	0.86-1.36	0.5099	0.02132
30	mir-224-5p	0.94	0.76-1.15	0.53984	0.02206
31	mir-375	1.05	0.9-1.21	0.55114	0.02279
32	mir-200b-3p	0.96	0.85-1.09	0.55515	0.02353
33	mir-92a-3p	0.93	0.74-1.18	0.55891	0.02426
34	mir-376c-3p	1.05	0.87-1.27	0.58603	0.025
35	mir-222-3p	0.93	0.71-1.21	0.58624	0.02574
36	mir-10a-5p	0.95	0.8-1.14	0.58751	0.02647
37	mir106b5p	0.93	0.72-1.2	0.5888	0.02721
38	mir-29a-3p	0.9	0.61-1.33	0.60193	0.02794
39	mir-20a-5p	0.94	0.73-1.21	0.62759	0.02868

40	mir-107	0.96	0.81-1.14	0.64153	0.02941
41	mir-372-3p	1.03	0.92-1.15	0.65346	0.03015
42	mir-145-5p	1.06	0.83-1.35	0.66189	0.03088
43	mir-19b-3p	1.05	0.84-1.32	0.67869	0.03162
44	mir-223-3p	0.97	0.84-1.13	0.71011	0.03235
45	mir-125b-5p	1.04	0.83-1.31	0.7311	0.03309
46	mir-25-3p	0.97	0.79-1.18	0.73265	0.03382
47	mir-10b-5p	1.03	0.88-1.19	0.74028	0.03456
48	mir-192-5p	1.03	0.85-1.24	0.75798	0.03529
49	mir-16-5p	1.03	0.84-1.27	0.76767	0.03603
50	mir15b5pe	0.96	0.7-1.3	0.77458	0.03676
51	mir-30e-5p	0.95	0.66-1.37	0.78088	0.0375
52	mir-30d-5p	0.96	0.71-1.29	0.78615	0.03824
53	mir-146a-5p	0.96	0.71-1.29	0.78791	0.03897
54	let7c-5p	1.03	0.83-1.28	0.80903	0.03971
55	mir-23a-3p	0.96	0.66-1.4	0.84597	0.04044
56	mir-214-3p	1.01	0.88-1.16	0.87316	0.04118
57	mir-124-3p	0.99	0.82-1.18	0.88939	0.04191
58	mir-423-5p	0.98	0.74-1.31	0.89365	0.04265
59	mir-210-3p	0.99	0.86-1.14	0.90323	0.04338
60	mir-885-5p	0.99	0.85-1.15	0.90989	0.04412
61	mir-75p	1.01	0.83-1.22	0.94637	0.04485
62	mir-19a-3p	1.01	0.82-1.23	0.94661	0.04559
63	mir-574-3p	1	0.87-1.15	0.95652	0.04632
64	mir-93-5p	1	0.79-1.28	0.96792	0.04706
65	mir-195-5p	1	0.81-1.23	0.96819	0.04779
66	mir-17-3p	1	0.87-1.15	0.97208	0.04853
67	mir-134-5p	1	0.88-1.14	0.98049	0.04926
68	mir-296-5p	1	0.87-1.15	0.99243	0.05

OS: overall survival; SCC: squamous cell carcinoma; HR: hazard ratio; 95% CI: 95% confidence interval; M: number of tests (68); δ: FDR level (0.05).

Table S4. miRNAs significantly associated with OS at univariable analysis for ADC patients.

**Rank (j)**	**Name**	**HR**	**95% CI**	***p*-value**	**(j/m)×δ**
1	mir-22-3p	1.29	1.05-1.59	0.01726	0.00074
2	mir-19b-3p	1.33	1.01-1.76	0.04331	0.00147
3	mir-195-5p	1.32	0.99-1.77	0.05904	0.00221
4	mir-26a-5p	0.73	0.51-1.04	0.08247	0.00294
5	mir-19a-3p	1.24	0.96-1.6	0.09548	0.00368
6	mir-18a-5p	1.31	0.95-1.81	0.09997	0.00441
7	mir-16-5p	1.24	0.94-1.64	0.12968	0.00515
8	mir-222-3p	1.25	0.93-1.67	0.13298	0.00588
9	mir-150-5p	0.86	0.69-1.06	0.15273	0.00662
10	mir-143-3p	1.14	0.94-1.38	0.16899	0.00735
11	mir-146a-5p	0.81	0.59-1.1	0.16992	0.00809
12	mir-193a-5p	1.18	0.93-1.51	0.1757	0.00882
13	mir-30d-5p	0.83	0.63-1.09	0.17684	0.00956
14	mir-93-5p	1.27	0.88-1.84	0.19379	0.01029
15	mir-17-3p	1.14	0.93-1.39	0.20068	0.01103
16	mir-574-3p	1.11	0.94-1.3	0.21273	0.01176
17	mir-29a-3p	1.22	0.87-1.7	0.24134	0.0125
18	mir-145-5p	1.14	0.91-1.43	0.26663	0.01324
19	mir-192-5p	1.09	0.93-1.29	0.27899	0.01397
20	mir-134-5p	1.08	0.93-1.26	0.317	0.01471
21	mir-204-5p	1.1	0.91-1.33	0.32739	0.01544
22	mir-26b-5p	0.88	0.65-1.18	0.38663	0.01618
23	mir-30e-5p	1.22	0.77-1.94	0.40646	0.01691

24	mir-296-5p	0.94	0.81-1.09	0.42031	0.01765
25	mir-27a-3p	0.85	0.57-1.27	0.42272	0.01838
26	mir-34a-5p	1.07	0.9-1.27	0.42838	0.01912
27	mir-31-5p	1.06	0.9-1.25	0.47091	0.01985
28	mir-128-3p	0.91	0.71-1.18	0.48108	0.02059
29	mir-885-5p	1.07	0.88-1.29	0.49004	0.02132
30	mir-92a-3p	0.91	0.7-1.19	0.49394	0.02206
31	mir-191-5p	0.91	0.69-1.21	0.50972	0.02279
32	mir-106b-5p	1.11	0.81-1.54	0.51482	0.02353
33	mir-75p	0.93	0.74-1.17	0.53883	0.02426
34	mir-373-3p	1.06	0.87-1.29	0.54591	0.025
35	mir-130b-3p	1.06	0.87-1.3	0.55281	0.02574
36	mir-15a-5p	0.93	0.71-1.2	0.55693	0.02647
37	mir-374a-5p	1.09	0.81-1.47	0.56552	0.02721
38	mir-10a-5p	0.94	0.76-1.16	0.56589	0.02794
39	mir-200c-3p	1.04	0.9-1.21	0.57195	0.02868
40	mir-107	0.95	0.8-1.13	0.57631	0.02941
41	mir-423-5p	0.93	0.71-1.22	0.60361	0.03015
42	mir-210-3p	0.96	0.81-1.15	0.66115	0.03088
43	mir-21-5p	0.92	0.61-1.37	0.67067	0.03162
44	mir-103a-3p	0.94	0.69-1.28	0.67991	0.03235
45	mir-122-5p	0.96	0.77-1.19	0.68486	0.03309
46	mir-200b-3p	1.04	0.87-1.23	0.68512	0.03382
47	mir-148a-3p	0.95	0.72-1.24	0.69138	0.03456
48	mir-214-3p	0.97	0.82-1.15	0.71291	0.03529
49	mir-196a-5p	1.02	0.91-1.15	0.76207	0.03603
50	mir-223-3p	0.98	0.86-1.12	0.77567	0.03676
51	let7a-5p	1.04	0.76-1.42	0.80015	0.0375
52	mir-155-5p	1.03	0.83-1.26	0.80892	0.03824
53	mir-224-5p	1.03	0.82-1.28	0.80982	0.03897
54	mir-375	0.98	0.81-1.18	0.81553	0.03971
55	mir-243p	1.03	0.78-1.38	0.81811	0.04044
56	mir-125b-5p	1.03	0.77-1.38	0.82834	0.04118
57	mir-23a-3p	0.98	0.79-1.21	0.84022	0.04191
58	mir-20a-5p	0.97	0.66-1.42	0.86103	0.04265
59	mir-211-5p	1.01	0.85-1.21	0.87306	0.04338
60	let7c-5p	1.02	0.79-1.31	0.8805	0.04412
61	mir-376c-3p	0.99	0.82-1.19	0.89854	0.04485
62	mir-10b-5p	1.01	0.83-1.22	0.91773	0.04559
63	mir-100-5p	0.99	0.76-1.28	0.92294	0.04632
64	mir-25-3p	0.99	0.75-1.3	0.93147	0.04706
65	mir-124-3p	1.01	0.81-1.25	0.95124	0.04779
66	mir-17-5p	1.01	0.72-1.42	0.96165	0.04853
67	mir-20-55p	1	0.86-1.16	0.97911	0.04926
68	mir-15b-5p	1	0.75-1.33	0.99196	0.05

OS: overall survival; ADC: adenocarcinoma; HR: hazard ratio; 95% CI: 95% confidence interval; m, number of tests (68); δ, FDR level (0.05).
